# Seeking truer measures of success: Moving toward more rigorous evaluations of industry-led access to medicines programs

**DOI:** 10.7189/jogh.11.03062

**Published:** 2021-05-15

**Authors:** Cherie Lynn Ramirez, Ashveena Gajeelee, Brianna Desharnais, Jenna Sherman, Dexter Waters

**Affiliations:** 1 Global Access in Action, Harvard University Berkman Klein Center for Internet & Society, Cambridge, Massachusetts, USA; 2 Simmons University, Boston, Massachusetts, USA; 3 Ariadne Labs, Brigham and Women’s Hospital and Harvard T.H. Chan School of Public Health, Boston, Massachusetts, USA; 4 Harvard T.H. Chan School of Public Health, Boston, Massachusetts, USA; 5 Thomas Jefferson University, Philadelphia, Pennsylvania, USA

Equitable access to essential medicines and diagnostic tools is crucial for improving global public health and promoting sustainable health development efforts. Many individuals suffering from health challenges around the world still do not have access to existing life-saving interventions due to lack of availability and high costs. The devastating effects of these inequities and discussions about their solutions have been at the forefront during the COVID-19 pandemic.

Health experts have long been working to identify effective methods of increasing access to medicines (AtM) that also incentivize research and development (R&D) by drug manufacturers. In the last two decades, the number of industry-led AtM programs implemented has substantially increased [1], with the pharmaceutical industry more openly recognizing a human rights obligation to improve access to medicines [2]. As argued by the *Lancet Commission on Essential Medicines Policies*, despite the existence of many industry-led AtM programs and the independent AtM Index that ranks 20 largest R&D-based pharmaceutical companies, rigorous evaluation remains a key challenge [3]. In response, the 2021 AtM Index report adapted various indicators to expand how it measures companies’ evaluation efforts and used tighter analytical framework centered on governance of access, research and development, and product delivery [4]. **Figure 1** illustrates a representative set of indicators that were changed between the 2018 and 2021 reports, with a full listing provided in Figure S1 in the **Online Supplementary Document**. This most recent report was released in January 2021 to assess the actions taken by pharmaceutical companies to expand access to medicine for people living in low- and middle-income countries. It also captures the industry’s response to the ongoing pandemic, identifying 63 new R&D projects in companies’ pipelines targeting COVID-19. The report also highlighted that the COVID-19 pandemic has caused further problems with funding competition for other AtM initiatives, such as to combat malaria, TB, and HIV/AIDS [5]. The Index reported that all 20 companies assessed now take steps to measure outcomes of AtM initiatives, up from 13 companies in 2018. Outcomes are made public for more than half of initiatives assessed (43 of 82). Also released in January 2021 was the Second World Health Organization (WHO) Model List of Essential In Vitro Diagnostics (EDL), which provides a comprehensive list of diagnostic tests necessary for universal health coverage that can be tailored to local circumstances [6]. The latest edition was revised to include COVID-19 nucleic acid amplification and antigen detecting tests as a necessary In Vitro Diagnostics. The WHO has previously worked with many countries to make noncommunicable disease diagnostic testing available in low- and middle-income countries, which has led to the development of resources like the National Free Diagnostic Service Initiative, which provides services in India [7]. Similar resources for COVID-19 are essential to stopping the spread of COVID-19 and improving global health.

**Figure 1:**
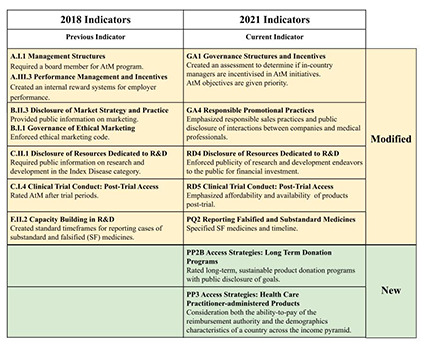
A small representative set of indicators are presented that were changed between the 2018 and 2021 Access to Medicines Index reports. A comprehensive listing of indicators and the changes between those used in 2018 and 2021 are provided in Figure S1 in the **Online Supplementary Document**.

In addition to the AtM Index, another major effort to improve reporting on AtM programs is Access Accelerated and its Access Observatory, which attempts not only to collect data, which is made publicly available, but also to devise a standardized measurement framework [8]. Their evaluation of pilot programs in the Philippines and Ghana suggests that differential pricing aided with health care strengthening can increase access to medicines [9].

A variety of factors continue to serve as barriers to conducting widespread, robust program evaluations, including the lack of specific data about drugs, quality medicine pricing data, and household usage of drugs. In addition, companies often lack incentives to collect and report high-quality impact data.

Despite these challenges, there are a small number of pharmaceutical companies that have conducted robust evaluations in the past. While none of the 120 AtM initiatives evaluated by Rockers et al. in 2017 met the high-quality threshold under the Grading of Recommendations, Assessment, Development and Evaluation (GRADE) system, three studies were classified as moderate quality: (1) Merck MSD’s Mectizan Donation Program, (2) the Pfizer International Trachoma Initiative, and (3) Novartis’s Access program in Kenya. The Novartis Access study was the first randomized assessment of the impact of a pharmaceutical industry-led AtM program, and initial results indicate that even offering medicines at US$1 per treatment per month did not significantly improve overall access nor reduce prices of the medicine after one year [10]. The study will be extended over a two-year period to test the factors which might have contributed to these results, which underscores the need for rigorous evaluations to understand and overcome barriers to access.

Program design varies widely between AtM initiatives, with the 120 AtM initiatives assessed in 2017 employing the following strategies: medicine donation (48%), price reduction (44%), licensing agreements (22%), and supply chain strengthening (11%). It has been found that bulk donations are more likely to be unsustainable and even cause harm in the long run by reducing competition [11]. General price reduction as a strategy alone may run the risk of not addressing other accessibility issues in a specific region. Increasing the quality of evaluations is necessary to determine the success of certain AtM strategies in a variety of contexts. More innovative, long-term approaches to improving AtM should also be explored, including differential pricing, voluntary licensing, and patent pooling [12]. Some of the metrics revised for the 2021 AtM Index report aim to better measure the scale and long-term sustainability of companies’ AtM strategies.

**Figure:**
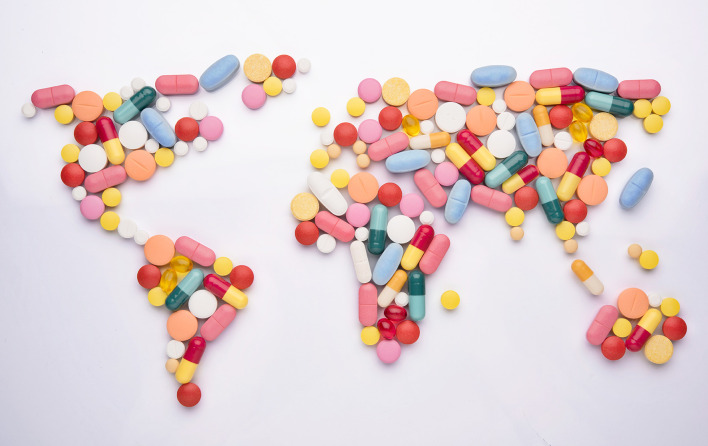
Photo: Image credit: ©pogonici/123RF.COM.

The urgent need to overcome COVID-19 has prioritized AtM among global leaders. In May 2020, the World Health Assembly adopted a resolution that calls for increased collaboration through “existing mechanisms for voluntary pooling and licensing of patents” to mitigate the COVID-19 pandemic and for “equitable access to and fair distribution of all essential health technologies and products to combat the novel coronavirus.” The resolution was co-sponsored by more than 130 countries and adopted by consensus [13]. Coupling expanded access with more rigorous and transparent evaluations will help ensure that best practices lead to desired outcomes and minimize unintended consequences. Fortunately, many actors across industry, academia, and public health recognize the need to prioritize equity, accountability, independence, and sound evidence. In doing so, companies can move toward strengthening their reputations while increasing meaningful impact that has the potential to save lives.

## Additional material

Online Supplementary Document
