# Supplementary material for: Seeking truer measures of success: Moving toward more rigorous evaluations of industry-led access to medicines programs
Source: J Glob Health. 2021 May 15;11:03062. doi: 10.7189/jogh.11.03062 (PMC8141331; doi:10.7189/jogh.11.03062)
Supplement: Online Supplementary Document [file jogh-11-03062-s001.pdf]

Supplementary Material:
Comparison of 2018 and 2021 Indicators for Access to Medicines Index

The indicators presented in this table are derived from the 2018 and 2021 Access to Medicines Index Reports as well as the Methodology report for the 2021 Access to Medicines Index Report.

Key:
Modified
New
Deleted
Retained

2018 Indicators	2021 Indicators	Changes
**A.I.1 Governance: Management structures** The company has a board member or board-level committee responsible for its access-to-medicine approach.<br>**A.III.3 Governance: Performance management & incentives** The company has internal incentive structures to reward the effective delivery of initiatives that improve access to medicine in countries within the Index scope, for diseases within the scope of the Index.	**GA1 Governance structures & incentives** The company has a governance system that includes direct board level responsibility and accountability for its access-to-medicine initiatives. To facilitate effective implementation of the strategy, senior management (i.e., CEO and/or senior executives) and in-country operational managers have access-to-medicine objectives and incentives to reward the effective delivery of initiatives that improve access to medicine in countries and for diseases within the Index scope.	**Modified** Indicators looking at governance structures and access-related incentives at the governance level have been merged. The indicator also newly assesses whether in-country managers are incentivised to meet objectives for access.
**A.I.2 Access-to-medicine strategy** The company sets objectives to improve access to medicine, and aligns its access-to-medicine strategy with its core business.	**GA2 Access-to-medicine strategy** The company has an access-to-medicine strategy and demonstrates that it is integrated within its corporate strategy. The strategy extends across the company's portfolio and pipeline, within the Index scope.	**Modified** The indicator newly covers how the company applies access thinking across its products and therapeutic areas.
**A.II.1 Managing for access-to-medicine outcomes: Public reporting** The company publicly reports on its commitments, objectives, targets and performance information related to improving access to medicine.	**GA3 Public disclosure of access-to-medicine outcomes** The company has time-bound measurable objectives, goals and targets related to improving access to medicine in countries in scope of the Index. It publicly shares progress against such objectives, goals and targets (i.e. outcomes).	**Retained**
**B.II.3 Disclosure of marketing strategy and practice** The company publicly discloses detailed information regarding its	**GA4 Responsible promotional practices** The company mitigates the risk of unethical sales practice (e.g. by	**Modified** Indicators assessing responsible sales practices and transparency on transfers of value to HCPs have

marketing and promotional programmes in countries within the Index scope (such as payments to or promotional activities directed at healthcare professionals and opinion leaders).<br><br>**B.I.1 Governance of ethical marketing**<br>The company commits to enforcing a code of conduct for ethical marketing practices that: extends to third parties; is consistent with existing industry standards; and incentivises responsible sales practice.	decoupling bonuses for sales agents from sales volumes only). Further, it takes a voluntary approach to publicly disclose information regarding actual transfers or its approach to transfers of value to healthcare professionals in countries in the Index scope (e.g. payments for attending and/or speaking at events, continuing medical education, promotional activities, or other non-monetary values directed at HCPs).	been merged.
**B.III.3 Compliance control: Internal control framework**<br>The company demonstrates that it has an internal control framework, which includes the following components:<br>a) fraud-specific risk assessment;<br>b) a monitoring system for compliance (other than auditing);<br>c) auditing and review mechanisms, which involve the use of both internal and external resources, apply to all third parties and all countries where it has operations, based on risk assessment;<br>d) procedures for segregation of duties between: management tasks and authorisation tasks; custody of assets and verification tasks; accounting tasks and payment tasks.	**GA5 Compliance Controls**<br>The company demonstrates that it has controls (internal controls, risk-based country audits, formal processes applying to third parties, etc.) in place to mitigate the risk of non-compliance in its operations in LMICs (i.e., in the areas of ethical marketing, anti-corruption and clinical trials).	**Modified**<br>The indicator assesses compliance controls where they respond to the specific needs of low- and middle-income countries.
**B.III.1 Ethical marketing and anti-corruption: Incidence of breaches**<br>The company has not been the subject of settled cases for corrupt practice or incidents of unethical marketing practice in countries within the scope of the Index during the past two years.<br><br>**C.III.7 Clinical trial conduct: Breaches**<br>The company has not been the subject of any breach of international codes	**GA6 Incidence of breaches**<br>The company has not been found to be the subject of negative legal rulings or settled cases for unethical marketing practices/ corrupt practices/ anti-competitive practices / misconduct in clinical trials in countries within the scope of the Index during the past two years.	**Modified**<br>All breaches will be assessed under one area of measurement, with the exception of anti-competitive behaviour related to influencing trade policy.

or lawsuits related to its clinical trial practices in countries within the scope of the Index during the last two years.<br><br>**E.III.6 Anti-competitive behaviour: No-IP**<br>There is evidence that the company has engaged in anti-competitive behaviour outside of its intellectual property strategy that impacts access to medicine.		
**E.II.1 Endorsement of TRIPS flexibilities**<br>The company publicly discloses its support of the policy flexibilities intended to protect public health confirmed by the Doha Declaration on TRIPS and Public Health.<br><br>**E.III.5 Anti-competitive behaviour: Trade policy Access to Medicine Index 2018**<br>There is evidence that the company employs an intellectual property (IP) strategy that is conducive to access to medicine, operating in accordance with the international consensus on IP standards as it pertains to public health, confirmed by the Doha Declaration.	**GA7 Trade policy: IP and access to medicine**<br>The company publicly discloses its support of the policy flexibilities intended to protect public health confirmed by the Doha Declaration on TRIPS and Public Health. Further, the company employs an intellectual property (IP) strategy that is conducive to access to medicine, operating in accordance with the international consensus on IP standards as it pertains to public health, confirmed by the Doha Declaration. This is evidenced by an absence of IP-related anti-competitive practices in relation to access to medicine in countries in scope.	**Modified**<br>Anti-competitive behaviour related to trade policy will be assessed alongside a company's publicly disclosed stance on the Doha Declaration on TRIPS and Public Health.
**C.III.2 R&D pipeline**<br>The size of the R&D pipeline within the scope of the Index, including innovative and adaptive R&D, and in-house and collaborative R&D.<br><br>**C.III.3 High-priority R&D**<br>The share of the company's R&D pipeline within the scope of the Index targeting specific needs of populations in countries also within the scope of the Index.	**RD1A R&D pipeline: Prioritised diseases**<br>The company engages in the development of products that target priority product gaps identified by global health research organisations. This includes both innovative and adaptive R&D and both in-house and collaborative R&D.	**Modified**<br>This indicator has been split into two indicators: for prioritised diseases and for other diseases.
**C.III.2 R&D pipeline**<br>The size of the R&D pipeline within the scope of the Index, including innovative and adaptive R&D, and in-house and collaborative R&D.	**RD1B R&D Pipeline: Other diseases**<br>The company engages in the development of products that address a clear public health need in low- and middle-income countries beyond the R&D priorities identified by global	**Modified**<br>This indicator has been split into two indicators: for prioritised diseases and for other diseases.

	health research organisations. This includes innovative and adaptive R&D that addresses, for example, heat stability issues and targets populations for which further studies/specific formulations are needed (such as children and pregnant women, etc.) as determined by stakeholder consensus.	
**C.I.2 Planning for access: Structured process**<br>The company has a process through which equitable access is planned for products successfully developed in-house and through R&D partnerships.	**RD2 Planning for access: Structured framework**<br>The company has a process through which equitable access is planned for products successfully developed both in-house and collaboratively.	**Retained**
**C.III.6 Planning for access: Project-specific plans**<br>The company provides evidence that its R&D projects (both in-house and collaborative) are supported by commitments and strategies to improve access to products that target diseases relevant to the Index in countries within the scope of the Index.	**RD3A Planning for access: Project-specific plans for prioritised diseases**<br>The company provides evidence that its R&D projects for diseases prioritised by WHO and the Policy Cures Research are supported by detailed commitments and strategies to improve access to products in countries within the scope of the Index.	**Modified**<br>This indicator has been split into two indicators: for prioritised diseases and for other diseases. Planning will newly take into account depth and quality of access plans, alongside breadth.
**C.III.6 Planning for access: Project-specific plans**<br>The company provides evidence that its R&D projects (both in-house and collaborative) are supported by commitments and strategies to improve access to products that target diseases relevant to the Index in countries within the scope of the Index.	**RD3B Planning for access: Project-specific plans for other diseases**<br>The company provides evidence that its R&D projects for diseases not prioritised by WHO and the Policy Cures Research are supported by detailed plans to improve access to products in countries within the scope of the Index.	**Modified**<br>This indicator has been split into two indicators: for prioritised diseases and for other diseases. Planning will newly take into account depth and quality of access plans, alongside breadth.
**C.II.1 Disclosure of resources dedicated to R&D**<br>The company publicly discloses the resources dedicated to its R&D activities conducted in-house and/or in collaboration for diseases within the scope of the Index and suitable for countries relevant to the Index.	**RD4 Disclosure of resources dedicated to R&D**<br>The company publicly discloses the resources dedicated to its R&D activities which are conducted in-house and/or in collaboration for diseases within the scope of the Index and suitable for countries relevant to the Index.	**Modified**<br>This indicator newly focuses solely on information companies place into the public domain.
**C.I.4 Clinical trial conduct: Post-trial access**	**RD5 Clinical trial conduct: Post-trial access**	**Modified**<br>This indicator newly expects not only

The company publicly commits to ensure post-trial access to treatments tested through clinical trials in countries within the scope of the Index.	The company has a publicly available policy on post-trial access that is aligned with the Declaration of Helsinki and includes a commitment to provide investigational treatments to all clinical trial participants who gain benefits from the treatment, where legally appropriate until the treatment is locally available and accessible. This commitment includes steps to register and considers affordability through reimbursement and access mechanisms in all countries where clinical trials have taken place.	registration, but also consideration of the affordability of products made available post-trial.
**F.III.2 Capacity building in R&D**<br>The company undertakes R&D capacity building initiatives in partnership with local universities and public sector research organisations that meet good practice standards in countries within the scope of the Index with the aim of increasing local capacity for health research (including clinical trial capacity) and product development.	**RD6 Capacity building in R&D**<br>The company increases local capacity for health research (including clinical trial capacity) and product development by undertaking R&D capacity building initiatives in partnership with local universities and public sector research organisations that meet Good Practice Standards in countries within the scope of the Index.	**Retained**
**D.III.4 Filing for marketing approval/registration: Needs-based**<br>The company has filed to register its newest products targeting diseases both within the scope of the Index scope in countries in need within scope.	**PR1 Registration**<br>The company rapidly files to register its most recently launched products targeting diseases within the scope of Index in countries within scope that have the highest disease burden.	**Modified**<br>The Index newly looks at not only the breadth of registration practice, but also the speed.
**D.III.1 Equitable pricing strategies: Market and product scope**<br>The company's equitable pricing strategies cover a significant percentage of the company's products that target diseases within the scope of the Index and a significant percentage of priority countries.<br>**E.III.1 Licensing: scale**<br>The company actively engages in issuing multiple voluntary licences and/or non-assert declarations for patented products within the Index scope, in countries within the Index scope.	**PP1 Access strategies: Coverage**<br>The company applies access strategies which aim to maximise patient reach across the selected products (e.g., equitable pricing strategies, voluntary licensing, non-assert declarations, donation programmes) in the greatest proportion of countries within the Index scope.	**New (but not ultimately used)**<br>* PP1 was deleted as comparisons were not possible with the data quality.

**G.I.1 Ad-hoc donation programmes**<br>The company has policies and processes in place to ensure ad-hoc donations are carried out in alignment with international guidelines and in response to an expressed need.	**PP2A Access strategies: Ad hoc donations**<br>The company has public policies and supply processes in place to ensure ad hoc donations are carried out rapidly in response to expressed need.	**Retained**
	**PP2B Access strategies: Long-term donation programmes**<br>The company engages in long-term, sustainable product donation programmes where elimination, eradication and control goals are possible, and publicly commits to the achievement of such goals.	**New**
**G.III.1 Quality of product donations**<br>The company and/or its partner(s) monitors the outcomes and impact of its structured donation programmes, and engages in capacity building activities to support the quality of the initiatives.<br><br>**G.III.2 Scale of product donations**<br>The number of countries and the number of beneficiaries reached through all of the company's structured donation programmes during the period of analysis.	**PP3 Access Strategies: Supranational products**<br>The company applies access strategies to the products it holds which are supranationally procured, through engaging with international procurers, advanced market commitments etc., and extends those strategies to countries graduating from development assistance or countries who do not qualify for such assistance.	**New**
**D.III.1 Equitable pricing strategies: Market and product scope**<br>The company's equitable pricing strategies cover a significant percentage of the company's products that target diseases within the scope of the Index and a significant percentage of priority countries.<br><br>**D.III.2 Equitable pricing strategies: Inter-country**<br>The company takes into consideration needs-based affordability and other relevant socioeconomic factors when making inter-country pricing decisions.<br><br>**D.III.3 Equitable pricing strategies: intra-country**<br>The company takes into consideration	**PP4 Access Strategies: Health Care Practitioner-administered Products**<br>The company takes into consideration both the ability-to-pay of the reimbursement authority and the demographics characteristics of a country in order to determine ability-to-pay of different segments of the country's population, aiming to increase reach for their healthcare practitioner-administered products across the income pyramid. This is evidenced by:<br>a) an approach which demonstrates how pricing strategies incorporate factors which determine payer's ability to pay for different segments of the population (e.g. patients	**New**

needs-based affordability and other relevant socioeconomic factors when making intra-country pricing decisions.	paying out of pocket) and non-pricing initiatives (i.e. patient assistance programmes, donations, voluntary licensing) complement those pricing strategies to maximise reach, and b) evidence of how the approach has increased the patient number since the product was introduced, and<br>c) plans to increase patient numbers for the following X years.	
**D.III.1 Equitable pricing strategies: Market and product scope**<br>The company's equitable pricing strategies cover a significant percentage of the company's products that target diseases within the scope of the Index and a significant percentage of priority countries.<br><br>**D.III.2 Equitable pricing strategies: Inter-country**<br>The company takes into consideration needs-based affordability and other relevant socioeconomic factors when making inter-country pricing decisions.<br><br>**D.III.3 Equitable pricing strategies: intra-country**<br>The company takes into consideration needs-based affordability and other relevant socioeconomic factors when making intra-country pricing decisions.	**PP5 Access Strategies: Self-administered products**<br>The company takes into consideration both the ability-to-pay of the reimbursement authority and the demographics characteristics of a country in order to determine ability-to-pay of different segments of the country's population, aiming to increase reach for their self-administered products across the income pyramid. This is evidenced by:<br>a) an approach which demonstrates how pricing strategies incorporate factors which determine payer's ability to pay for different segments of the population (e.g. patients paying out of pocket) and non-pricing initiatives (i.e. patient assistance programs, donations, voluntary licensing) complement those pricing strategies to maximize reach, and b) evidence of how the approach has increased the patient number since the product was introduced, and<br>c) plans to increase patient numbers for the following X years.	**New**
**E.I.1 Patent filing and enforcement**<br>The company publicly commits to not filing for or enforcing patents related to diseases within the Index scope in Least Developed Countries, low income countries, and a subset of lower-middle income countries and upper-middle income countries.	**PPL1 Patent filing & enforcement**<br>The company publicly commits to not filing for or enforcing patents related to diseases within the Index scope in Least Developed Countries, low-income countries, and a subset of lower-middle income countries and upper-middle income countries.	**Retained**

**E.II.2 Patent disclosure**<br>The company publicly discloses the patent status of its products for diseases relevant to the Index, in countries within the Index scope.	**PPL2 Patent status disclosure**<br>The company publicly discloses the patent status of its products for diseases relevant to the Index, in countries within the Index scope.	**Retained**
**E.III.2 IP sharing**<br>The company provides evidence of sharing its intellectual capital (e.g., molecules library, patented compounds, processes or technologies) with research institutions and neglected disease drug discovery initiatives (e.g., WIPO Re: Search, Conserved Domain Database (CDD), Open Source Drug Discovery (OSDD)) that develop products for diseases relevant to the Index on terms conducive to access to medicine for countries within the scope of the Index.	**PPL3 IP Sharing**<br>The company provides evidence of sharing its intellectual capital (e.g., molecules library, patented compounds, processes or technologies) with research institutions and neglected disease drug discovery initiatives (e.g., WIPO Re: Search, Conserved Domain Database (CDD), Open Source Drug Discovery (OSDD)) that develop products for diseases relevant to the Index on terms conducive to access to medicine for countries within the scope of the Index.	**Retained**
**E.II.3 Disclosure of licensing practice**<br>The company publicly discloses detailed information about the voluntary licences and non-assert agreements it is engaged in, for products within the Index scope, in countries within the Index scope.<br>**E.III.3 Access-oriented licensing**<br>The company includes access-oriented terms and conditions within the voluntary licences it agrees for products relevant to the Index, in countries within the Index scope.	**PPL4 Access-oriented quality licensing**<br>The company agrees access-oriented, transparent non-exclusive voluntary licences which include clauses that facilitate affordability and supply of quality products.	**Retained**
**E.III.4 Licensing: Geographic scope**<br>The company includes a broad range of countries within the geographic scope of its licences, including middle-income countries outside of sub-Saharan Africa with comparatively high burdens of disease.	**PPL5 Licensing: Geographic scope**<br>The company includes a broad range of countries within the geographic scope of its licences, including middle-income countries outside of sub-Saharan Africa with high burdens of disease.	**Retained**
**D.III.7 Aligning supply and demand**<br>The company makes efforts to	**PQ1 Ensuring continuous supply**<br>The company has processes in place to improve supply chain efficiency	**Modified**<br>This indicator has been made to better capture actions taken by companies to

understand product distribution and demand behaviour in countries in the scope of the Index beyond first product hand-off, and takes informed action to ensure products are made available in sufficient quantities in a timely manner.	for all its product within the Index scope, making efforts to understand product distribution and demand behaviour in countries in the scope of the Index beyond first product hand-off, takes informed action to ensure uninterrupted supply and making products available in sufficient quantities in a timely manner. This process includes the following elements: a) has an established forecasting/information systems to manage its supply chain<br>b) manages a safety stock of relevant products<br>c) works with several API suppliers to prevent shortages<br>d) communicates plans with governmental agencies, regulators, purchasers, hospitals and other relevant stakeholders to align demand and supply<br>e) works with other collaborators on managing stockouts and shortages<br>f) ensures supply in at least one Least Developed Country.	ensure uninterrupted supply.
**F.II.2 Supply chain management: Reporting falsified and substandard medicines**<br>The company has a policy/protocol for reporting sub-standard and falsified (SF) medicines in countries within the scope of the Index that specifies timeframes for reporting to relevant stakeholders (i.e., national regulatory authorities and WHO Rapid Alert).	**PQ2 Reporting substandard and falsified medicines**<br>The company has a policy/protocol for reporting substandard and falsified (SF) medicines in countries within the scope of the Index that specifies timeframes for reporting to relevant stakeholders (i.e., national regulatory authorities and WHO Rapid Alert).	**Modified**<br>The Index has clarified what a confirmed case of SF medicines is (i.e, if confirmation can take place by visual inspection) and made slight adjustments to the relevant timelines.
**F.III.1 Capacity building in manufacturing**<br>The company undertakes manufacturing capacity building initiatives with local manufacturers aimed at achieving international Good Manufacturing Practice (GMP). These initiatives meet good practice standards* in countries within the scope of the Index.	**PCB1 Capacity building in manufacturing**<br>The company undertakes manufacturing capacity building initiatives with local manufacturers aimed at achieving international Good Manufacturing Practice (GMP). These initiatives meet good practice standards* in countries within the scope of the Index.	**Retained**
**F.III.3 Capacity building in supply**	**PCB2 Capacity building in supply**	**Retained**

**chain management**<br>The company undertakes supply chain capacity building initiatives in countries within the scope of the Index in partnership with local stakeholders (e.g., ministries of health, procurement, logistics and distribution agencies) that meet good practice standards with the aim of improving the affordability, accessibility and quality of products.	**chain management**<br>The company undertakes supply chain capacity building initiatives in countries within the scope of the Index in partnership with local stakeholders (e.g., ministries of health, procurement, logistics and distribution agencies) that meet Good Practice Standards with the aim of improving the affordability, accessibility and quality of products.	
**F.III.5 Health system strengthening**<br>The company undertakes health system strengthening initiatives related to access to medicine in partnerships with local stakeholders (where there is no conflict of interest) that meet good practice standards in countries within the scope of the Index	**PCB3 Health System Strengthening**<br>The company undertakes health system strengthening initiatives in partnership with local stakeholders (where there is no conflict of interest) that meet Good Practice Standards in countries within the scope of the Index. Such initiatives should work in a coordinated way with other parties, complementing the local health system, with outcomes clearly monitored.	**Modified**<br>The Index newly looks for greater coordination and integration into local systems, and stakeholders now expect the measurement of outcomes as a basic requirement.
**A.IV.1 Innovation in business models**<br>The company has contributed to the development of innovative business models that meet the access needs of patients in countries within the Index scope.	**PBM1 Inclusive business models**<br>The company has contributed to the development and implementation of scalable inclusive business models that aim to meet the access needs of populations at the base of the income pyramid (which may include vulnerable populations) in countries within the Index scope, with a long-term horizon.	**Modified**<br>The Index newly covers business models targeting vulnerable populations, alongside models targeting the base of the income pyramid. Pilots will still be assessed, these should be scalable, with successfully scaled projects being the highest standard.
**A.II.2 Stakeholder engagement: Public reporting** The company publicly discloses summaries of: its stakeholder selection process; stakeholder groups it engages with; engagement activities related to access to medicine; and key outcomes and rationales.		**Removed**
**A.III.1 Managing for access-to-medicine outcomes: Performance management system**<br>The company has a performance management system to monitor and measure the outcomes and impact of		**Removed**

its access-to-medicine activities across its global operations.		
**A.III.2 Stakeholder engagement**<br>The company engages with relevant stakeholders, including universities, industry peers, patient groups, local governments, employees, and local and international non-governmental organisations, with the aim of improving access to medicine. The company has a system in place to incorporate local and other external perspectives on access-to-medicine in the development and implementation of its access strategies.		**Removed**
**A.IV.2 Innovation in governance and stakeholder engagement**<br>The company has developed innovative (unique in the sector) approaches to its access governance, its performance management systems and/or its stakeholder engagement.		**Removed**
**B.I.2 Governance of Anti-Corruption**<br>The company commits to proactively engaging in addressing corruption through: its internal policies, oversight of third parties, external commitments; and memberships.		**Removed**
**B.II.1 Market influence: Policy positions**<br>The company is transparent about political contributions made, and the policy positions it seeks to promote that have an impact on access to medicine in countries within the scope of the Index.		**Removed**
**B.II.2 Market influence: Memberships**<br>The company publicly discloses board seats and memberships held, and financial support provided to organisations through which it may advocate policies relevant to access to medicine in countries within the Index scope. The company also discloses policies for responsible		**Removed**

engagement and management of conflicts of interest.		
**B.II.4 Ethical marketing and corruption: Disclosure of breaches** The company publicly discloses information regarding breaches in countries within the scope of the Index of internationally recognised codes of conduct, laws and regulations that govern ethical marketing and corruption in the last two years.		**Removed**
**B.III.2 Ethical marketing and anti-corruption: Enforcement** The company has clearly defined enforcement procedures and (where there has been misconduct) provides evidence of taking disciplinary action against employees or third parties who have violated its code of conduct for ethical marketing or anti-corruption. The company provides evidence of follow-up actions taken to mitigate the risk of future breaches.		**Removed**
**B.IV.1 Innovation in market influence and compliance** The company has adopted an innovative approach to improving ethical business performance in countries within the scope of the Index relating to ethical marketing, responsible lobbying, and anti-corruption.		**Removed**
**C.I.1 Product development: R&D commitment and strategy** The company publicly commits to conduct R&D of products for diseases within the scope of the Index with the goal of improving access to medicine in countries within scope. It operationalises its commitments with an R&D strategy that takes public health needs into account and has a system for setting targets and evaluating progress over time.		**Removed**
**C.I.3 Clinical trial conduct: Policies and compliance systems**		**Removed**

The company commits to and has processes to ensure compliance with standards of quality assurance, control and ethics when conducting clinical trials in countries within the Index scope. These standards are consistent with codes such as Good Clinical Practice (GCP), and the Declaration of Helsinki, regardless of whether the trials are conducted in-house or through a third-party, e.g., contract research organisation (CRO).		
**C.III.1 Resources dedicated to R&D** The financial R&D investment dedicated to diseases within the scope of the Index out of the company's total revenue.		**Removed**
**C.III.4 Collaborative R&D: Share of pipeline** The share of the company's research pipeline (both innovative and adaptive) within the scope of the Index that is being developed in partnership.		**Removed**
**C.III.5 Product development: Movement through the pipeline** The number of candidates relating to diseases within the scope of the Index moving through the R&D life cycle from early research phases to more advanced phases.		**Removed**
**C.IV.1 Innovation in R&D** The company has adopted innovative (unique in the sector), sustainable or open business models to further the global R&D agenda for the development of products for diseases relevant to the Index.		**Removed**
**D.I.1 Commitment to equitable pricing** The company publicly commits to implementing equitable pricing strategies for its products for diseases within the Index scope, in countries within scope.		

**D.I.2 Filing for marketing approval/registration targets**<br>The company commits to filing for marketing approval or product registration within a specific timeframe in sub-Saharan Africa and low-income countries for products for diseases within the scope of the Index, considering public health need.		**Removed**
**D.II.1 Equitable pricing strategies: Volume of sales disclosure**<br>The company discloses the volume of sales for products covered under equitable pricing programmes within the scope of the Index.		**Removed**
**D.II.2 Equitable pricing strategies: Price disclosure**<br>The company discloses ex-manufacturer prices for products covered under equitable pricing programmes within the scope of the Index.		**Removed**
**D.II.3 Public disclosure of registration status**<br>The company publicly discloses the status of marketing approvals for products in countries in scope.		**Removed**
**D.III.5 Drug recall system**<br>The company has in place policies and processes, procedures and resources needed to carry out effective drug recalls (product and packaging) in countries within the scope of the Index, and provides details of its recall system effectiveness.		**Removed**
**D.III.6 Brochure and packaging adaptation: Rationale use**<br>The company provides evidence of needs-based brochure and packaging adaptations to facilitate rational use for its products destined for countries within the scope of the Index.		**Removed**
**D.IV.1 Innovation in Pricing, Manufacturing and Distribution**<br>The company has introduced		**Removed**

innovative approaches (unique in the sector) to equitable pricing, manufacturing and distribution that help with sustainable delivery of products for diseases within the Index scope to individuals in the countries relevant to the Index. If the approach focuses on equitable pricing, it targets those who face the highest financial barriers to access.		
**E.IV.1 Innovation in Patents & Licensing**<br>The company has adopted innovative (unique in sector) programmes aimed at managing the exclusivity conferred by patent protection to support competition for products relevant to the Index, in countries within the Index scope.		**Removed**
**F.II.1 Pharmacovigilance: Sharing safety data**<br>The company shares post-marketing surveillance data with relevant authorities beyond legal requirements and updates product safety and/or efficacy labels (regardless of product life cycle stage) in countries within the scope of the Index.		**Removed**
**F.III.4 Capacity building in pharmacovigilance**<br>The company undertakes pharmacovigilance capacity building initiatives with reputable partners that meet good practice standards with the aim of developing and strengthening national pharmacovigilance systems in countries within the scope of the Index.		**Removed**
**F.IV.1 Innovation in Capacity Building**<br>The company has developed or adopted innovative (i.e., unique in sector) approaches to building capacity related to access to medicine through partnerships with relevant stakeholders in countries within the scope of the Index.		**Removed**

**G.III.1 Quality of product donations**<br>The company and/or its partner(s) monitors the outcomes and impact of its structured donation programmes, and engages in capacity building activities to support the quality of the initiatives.		**Removed**
